# Correspondence to the article by Wang et al., “Association between blood pressure and the risk of biopsy-induced endobronchial hemorrhage during bronchoscopy”

**DOI:** 10.1186/s12890-023-02330-w

**Published:** 2023-03-08

**Authors:** Vinod Govindasaami

**Affiliations:** Sri Ramachandra University, Chennai, India

I read with great interest the article “Association between blood pressure and the risk of biopsy-induced endobronchial hemorrhage during bronchoscopy” by Wang et al., It provides great insight into the hypertension and bleeding risk in a specific category of bronchoscopy procedure—endobronchial biopsy, in a specific population—lung malignancy. But I would like to offer the following comments.

- Pulmonary hypertension and congestive heart failure are significant factors for bleeding during bronchoscopic biopsies which have not been considered for the present study. [1]
- In the analysis, previous history of hemoptysis prior to bronchoscopy was not included which has been noted to be a predictor of hemoptysis probably as it suggests the tumour to be more vascular. [2]
- The location of the lesion in proximity to a vessel (like superior venacava syndrome)/vascularity of the lesion were not considered for the analysis of bleeding which might be a significant confounder.
- All the bronchoscopies are done under General Anesthesia (GA) which is not recommended routinely and guidelines recommend that flexible bronchoscopic biopsies can be safely done under local anesthesia/mild sedation using midazolam. Being performed under GA, the bleeding risk may not be comparable to doing under LA/mild sedation. [3]
- Finally, the bleeding occurred in the study population were all non-severe hemorrhage that was successfully controlled by bronchoscopy itself and didn’t lead to adverse outcome of any patient or lead to further procedures for bleeding control—and hence might not affect the Bronchoscopists to change their practice but might pique the interest for researchers for further prospective studies.
- Also it would be of great interest to know in how many patients the bleeding affected the histopathological diagnosis needing a second procedure for obtaining tissue diagnosis.

## Data Availability

Not applicable.
